# Efficacy of Fiber-Enriched Versus Fiber-Free Enteral Feeds on Bowel Function of Non-Critically Ill Tube-Fed Adult Patients in Saudi Arabia—A Prospective Cohort Study

**DOI:** 10.3390/nu17040676

**Published:** 2025-02-13

**Authors:** Mostafa A. Abolfotouh, Rawan A. Alolayan, Heba Binhusain, Abdulrahman Alsayegh, Ibrahim T. Al Babtain

**Affiliations:** 1 King Abdullah International Medical Research Center, King Saud bin Abdulaziz University for Health Sciences, Ministry of National Guard Health Affairs, Riyadh 11426, Saudi Arabia; albabtainib@ngha.med.sa; 2 Family Health Department, High Institute of Public Health, Alexandria University, Alexandria 21544, Egypt; 3 Department of Clinical Nutrition, King Abdulaziz Medical City, Ministry of National Guard Health Affairs, Riyadh 11426, Saudi Arabia; alolayanra@ngha.med.sa (R.A.A.); binhusainhe@mngha.med.sa (H.B.); 4 College of Applied Medical Sciences, King Saud University, Riyadh 11362, Saudi Arabia; afs1914@gmail.com; 5 Department of Surgery, King Abdulaziz Medical City, Ministry of National Guard Health Affairs, Riyadh 11426, Saudi Arabia

**Keywords:** dietary fiber, fiber supplementation, diarrhea, enteral feeding, bowel function, significant weight loss, absolute risk reduction, relative risk reduction, Saudi Arabia

## Abstract

Background: There is controversy regarding whether using fiber-enriched formula affects the incidence of diarrhea among enterally fed patients in our setting. Also, there is a lack of clinical studies about enterally fed patients’ tolerance for feeding and the incidence of diarrhea among patients in the Middle East. This study aimed to assess fiber enrichment’s efficacy in reducing post-enteral feeding bowel intolerance in non-critically ill patients. Methods: This was a prospective cohort study of 55 fiber-free (FF) and 119 fiber-enriched (FE) tube-fed adult patients admitted for five or more days with medical or surgical conditions. Data on patients’ demographics, antibiotics and laxative medications, and gastrointestinal complications were collected. Absolute risk reduction (ARR), relative risk reduction (RRR), and relative risks (RR) were calculated to assess the efficacy of fiber enrichment in reducing post-enteral feeding bowel intolerance. Statistical significance was set at *p* ≤ 0.05. Results: The rate of diarrhea dropped from 54.5% for FF to 29% for FE groups, with an ARR of 25.1% (95% CI 24.6–25.6, *p* < 0.001) and an RRR of 64.1%, and RR was 0.54, reflecting a reduction in the rate of diarrhea by 46% after fiber enrichment. The rate of significant weight loss dropped from 45.5% without enrichment to only 26.9% with enrichment, with an ARR of 18.6% (95% CI: 18.0–19.2, *p* < 0.001) and RRR of 40.9%, and RR was 0.59, reflecting a 41% reduction in significant weight loss after fiber enrichment. After adjusting for some potential confounders, FF formula was a significant predictor of diarrhea (OR = 3.04, 95% CI 1.49–6.19, *p* = 0.002) and significant weight loss (OR = 2.37, 95% CI 1.16–4.84, *p* = 0.018) in tube feeding, while antibiotic intake was also a significant predictor of only diarrhea (OR = 2.68, 95% CI 1.12–6.38, *p* = 0.026). Conclusions: This study demonstrated the beneficial effect of fiber supplementation in minimizing diarrhea in hospitalized patients receiving tube feeding. Antibiotic usage must be scrutinized and stopped if possible. Overall, the study provides compelling evidence supporting fiber-enriched enteral feeding, though further discussion on potential confounders and clinical applications would enhance its impact. Further, well-designed RCTs are needed to prove the efficacy of fiber-enriched feeds used in enteral tube feeding in non-critically ill patients.

## 1. Introduction

Enteral nutrition (EN) is a common technique used to support patients when oral food intake is inadequate, and the patients cannot meet their nutrition needs. The European Society for Enteral and Parenteral Nutrition (ESPEN) advises beginning early tube feeding (within 24 h) in patients for whom early oral intake cannot be started and in whom oral consumption will be insufficient (less than 50%) for greater than 7 days. Special risk populations are patients undergoing primary head and neck or gastrointestinal surgery for cancer, patients with intense trauma with brain damage, and patients with apparent malnutrition at the time of surgery [1].

Diarrhea is one of the highest side effects associated with tube feeding. Clinical research shows that 18–32% of hospitalized enterally fed patients in medical and surgical wards get diarrhea [2,3]. The prevalence of diarrhea depends on its operational definition [4], as many definitions have been observed by Leak et al. [5]. Diarrhea can cause electrolyte disturbance, dehydration, increased patient discomfort, increased risk of wounds, fecal contamination, increased nursing workload, and enteral feeding cessation that may lead to malnutrition. In addition, malnutrition increases the patient risk of complications, a chief reason for readmission, doubled the risk of pressure ulcers [6,7], increases mortality, increases patient falls by 45% [8], increases the length of hospital stay [9], and increases health care costs by more than 60% [10].

The pathogenesis of diarrhea during enteral feeding is multi-factorial, including antibiotics, colonic water secretion, and enteropathogenic infection. The onset of altered physiological responses may be observed during EN. Studies on healthful people discovered that intragastric enteral feeding results in atypical water secretion into the ascending colon [11]. This can be aggravated by suppressing distal colonic motor motion that speeds up colonic transit and decreases the chance for water absorption [12]. If these happen in patients receiving enteral feeding, diarrhea might also occur without absorptive mechanisms. On the other hand, antibiotic-associated diarrhea occurs in about 5–30% of patients either early during antibiotic therapy or up to two months after the end of the treatment [13], through disturbing the gastrointestinal (GI) microbiota. Several other mechanisms for diarrhea during enteral tube feeding have been established, including medications such as antibiotics and laxatives, tube feeding applications, and nature (formula temperature, osmolality, and flow rate) [14,15]. Also, fecal impaction may be confused with diarrhea [16]. Strict guidelines for these characteristics of enteral solutions have not been made [17].

Several ways have been used to manage diarrhea in enteral feeding: prebiotics, probiotics, FODMAPs, fibers, and semi-elemental formula. Semi-elemental formulas are nutritionally complete pre-digested formulas that provide protein as amino acids or small peptides, fat primarily as medium-chain triglycerides, and carbohydrates as fructose or maltodextrin. Prebiotics are “selectively fermented substances that permit precise modifications, each in the composition and/or action inside the gastrointestinal microflora that confer advantages upon host health.” Prebiotics resist the digestive enzymes in the gut but get fermented by the colonic microflora, bifidogenic and pH-lowering effects [18,19], and this inhibits potentially pathogenic bacteria [20]. Probiotics are “live microorganisms that give health benefits to the host when ingested in sufficient quantities” [21]. Probiotics integrate with the microflora of the host or immunocompetent cells of the intestinal mucosa. FODMAPs (fermentable, oligosaccharides, disaccharides, monosaccharides, and polyols) are short-chain carbohydrates that are inefficiently absorbed in the small intestine before travelling to the large intestine where bacteria rapidly ferment them [22], which may result in taxation and distension of the intestine inducing bloating, abdominal colic, and increased water transit (osmotic impact), which can alter gut motility and promote diarrhea [23]. Restricting dietary FODMAPs has been shown to improve GI symptoms. FODMAPs are added to the EN formula as fructo-oligosaccharides (FOS) and inulin [24].

Dietary fiber is mostly a carbohydrate that human digestive enzymes cannot digest [25]. Intestine microflorae can ferment dietary fibers into methane, hydrogen, CO2, and short-chain fatty acids. Short-chain fatty acids, a main essential energy substance for colon epithelial mucosa, is vital for the regeneration of epithelia, enhancing absorption of H_2_O and Na, regulating the function of the bowel [26,27,28], protecting the intestinal barrier, and preventing bacterial translocation. One systematic review on the clinical role of fiber-enriched enteral formulae identified 25 studies that showed no difference in bowel movement frequency when the formula was administered with or without fiber [29]. Meanwhile, randomized controlled trials (RCTs) to compare fiber-enriched formula and fiber-free formula in five studies showed no significant reduction in the incidence of diarrhea [29]. However, Elia et al. [30]. conducted a systematic review and meta-analysis of fiber-containing enteral formulae, 51 studies, including 43 RCTs in adults and children receiving EN; the results showed significant benefits of fiber-supplemented enteral formulas for patients with diarrhea, particularly in those with a high baseline incidence of diarrhea. Kamarul et al. [31] conducted a systematic review and meta-analysis of fiber-supplemented EN in twenty-six studies conducted in adults; the results showed that fiber helps minimize diarrhea in patients receiving EN, particularly in non-critically ill patients.

There is controversy regarding whether using fiber-enriched formula affects the incidence of diarrhea among enterally fed patients in our setting. Also, in the Middle East, there is a lack of clinical studies about enterally fed patients’ tolerance for feeding and the incidence of diarrhea among patients. Studies have been conducted on enteral feeding among critically ill patients at King Abdulaziz Medical City (KAMC) of the Ministry of National Guard Health Affairs (MNGHA) in Saudi Arabia [32,33,34,35,36]. No such studies were conducted among non-critically ill patients. Thus, the main aim of this study was to assess the efficacy of fiber-enrichment of tube feeding on the reduction in post-enteral feeding bowel intolerance among non-critically ill adult patients in Saudi Arabia to optimize hospital resources and regulate formula prescriptions.

## 2. Methods

### 2.1. Study Setting

This study was conducted on surgical and medical inpatients at King Abdulaziz Medical City, Ministry of National Guard (KAMC)—Health Affairs, in Riyadh- Central region. KAMC is a major tertiary care institution serving patients referred throughout the Kingdom of Saudi Arabia, with a capacity of 1200 beds. Surgical and medical units are accommodating more than 700 patients/month. The clinical nutrition department, with specialized clinical dietitians, provides specific nutritional care services and counseling to both in- and out-patients and supervises patients’ food services.

Enteral feeding delivers a nutritionally complete feed (containing protein, carbohydrates, fat, water, minerals, and vitamins) directly into the gut via a tube. About 10–15% of medical and surgical patients in KAMC are on fiber or fiber-free formulas (around 100 patients/month). Fiber-free (FF) formula (Ensure) is balanced and complete nutrition for long-term tube feeding, isotonic, lactose and gluten-free, and low-residue. Fiber-enriched (FE) formula (Jevity) is balanced and complete nutrition for long-term tube feeding, isotonic, lactose, and gluten-free. Fiber-fortified formula helps maintain normal bowel function. These are from the same manufacturing company [Abbott Laboratories, Illinois, United States]. Table 1 shows the contents of Ensure and Jevity formulas per 1000 calories. We start with the isotonic formula, continuous feeding at 50 mL/h, and increase it by 30 mL every 4 h until we reach the volume that a clinical dietitian indicates. After 2 days, if the patient tolerates the feeding well, we switch to intermittent feeding, starting at 100 mL every 4 h and increasing it by 50 mL every 4 h until reaching the volume indicated by a clinical dietitian.

### 2.2. Study Subjects

The study comprised adult and geriatric patients of both genders admitted to the hospital with non-critical medical or surgical illnesses, whose early oral intake could not begin and whose oral consumption would be insufficient (less than 50%) for more than 7 days. Patients on exclusive enteral tube feeding, either fiber-free or fiber-enriched feeding formulas based on the treating dietitian’s preferences, were made the target of the study. The exclusion criteria were: (1) Patients in GI radiation therapy, chemotherapy, contraindications for enteral feeding (gastrointestinal tract obstruction, hemorrhage and ileus), bowel resection and anastomosis, enteric fistula, pancreatitis, gastrointestinal diseases (ulcerative colitis, Chron’s disease, and ischemic or infectious colitis), immunosuppressed patients, (2) Patients with diarrhea occurring within 72 h before admission, (3) Patients on EN for less than five consecutive days, and (4) ICU patients on enteral feeding, to avoid being confounded with patients with severity scales different from those in the wards.

### 2.3. Study Design

This study has a prospective cohort design, and every enterally fed patient was followed for 5 to 14 days from the start date of enteral feeding.

### 2.4. Sample Size and Sampling Technique

Based upon an average incidence of diarrhea of 25% in previous epidemiological studies of hospitalized enterally fed patients in medical and surgical wards [2,3], with the assumption of an expected incidence of diarrhea of 8% among those on fiber enrich (FE) formula, 95% confidence interval, and 80% power, and FF:FE ratio of 1:2, the estimated sample sizes were 104 patients on fiber-enriched formula and 52 patients on fiber-free formula. A total of 174 patients were followed up in the study (119 FE group and 55 FF group). All patients who fit the inclusion criteria were chosen from both groups after consenting and followed up for 5 to 14 days until the final sample sizes were fulfilled as convenient samples.

### 2.5. Data Collection

In this study, all patients in surgical and medical wards who were on enteral feeding were observed daily, from the first day of the delivered formula or for 5–14 days based on their length of stay, to determine the incidence of diarrhea and its frequency, in both groups (fiber enriched and fiber-free formula). The following data were collected:

1. Patient’s characteristics: age, gender, BMI (on the first and last day of enteral feeding), ICU admission. Each patient’s Charcot comorbidity score (CCI) was calculated [37,38].

2. Enteral feeding:

a Type of enteral feeding: Fiber-free formula or fiber-enriched formula.
b Volume ratio (Percent of EN volume ratio): The efficiency of EN was measured daily by volume ratio (%): VR (%) = (administered volume of nutrition/prescribed volume of nutrition) × 100. Energy requirements were calculated using the Harris–Benedict equation [39], and the protein requirement for surgical patients was 1.2–1.5 g/kg. The dietitian calculated the volume of the enteral formula prescribed for each patient based on the patient’s energy requirement.
c Underweight. Underweight was considered for ≥65-year-old patients whose initial BMI was <22 kg/m^2^, or for <65-year-old patients whose initial BMI was <18.5 kg/m^2^.
d Antibiotics and laxative medications. Laxatives [Docusate, Glycerin rectal, Magnesium Sulphate, Bisacodyl Suppository, Fleet enema, Movicol, Lactolose, Prune juice, milk of magnesia, senna]. Antibiotics [Tigecycline, Meropenem, Tazocin, Augmentin, Cefepime, Ceftriaxone, Moxifloxacin, Clindamycin, Co-trimoxazole].
e Other medications, e.g., nonsteroidal anti-inflammatory drugs, alpha-glucosidase inhibitors, antineoplastics, magnesium-based antacids, colchicine sorbitol- or -lactose-based drugs, prostaglandins, antiarrhythmic drugs, cholinergic drugs, anthraquinone-related drugs, lipase inhibitors, and cholinesterase inhibitors.

3. GI intolerance: GI complications were defined as follows [40]:

a Gastric residual is considered high if the volume exceeds 500 mL.
b Regurgitation is defined as observing the enteral formula in the oral cavity.
c Vomiting will be defined as the return of the enteral formula from the stomach to the mouth.
d Abdominal distention is the presence of excessive gases in the stomach or intestine. It is diagnosed with tympany, or the absence of bowel sounds during physical examination.
e Constipation is the absence of bowel motion for more than three days.
f Diarrhea is defined as a daily accumulation score of more than or equal to 12, as proposed by Hart and Dobb [41]. This is aligned with our practice at the Ministry of National Guard. The nursing staff and the study investigators collected scores daily. Patients were followed daily for 14 successive days unless discharged or in cases of death.
g Significant weight loss was considered as a loss of ≥2% of the initial body weight over the study period.

### 2.6. Data Analysis

Descriptive analyses of mean, standard deviation, and frequency (%) were applied. Analytical statistics, such as t-tests, were used to compare the incidence of diarrhea among patients on tubes fed with fiber-enriched and those with fiber-free formulas. Relative risk and corresponding confidence intervals were calculated for each factor associated with diarrhea incidence. Regression models were applied to adjust for confounders, such as antibiotic and laxative medication, in the association between the type of formula (FF or FE) and the incidence of complications (diarrhea and significant weight loss). Both absolute risk reduction (ARR) and relative risk reduction (RRR) were calculated as follows: ARR = Incidence of diarrhea with no fiber enriched − Incidence of diarrhea with fiber-enriched, RRR = (Incidence of diarrhea with no fiber enriched − Incidence of diarrhea with fiber-enriched)/Incidence of diarrhea with no fiber-enriched. Statistical significance was set at *p* ≤ 0.05.

## 3. Results

**Figure 1:**
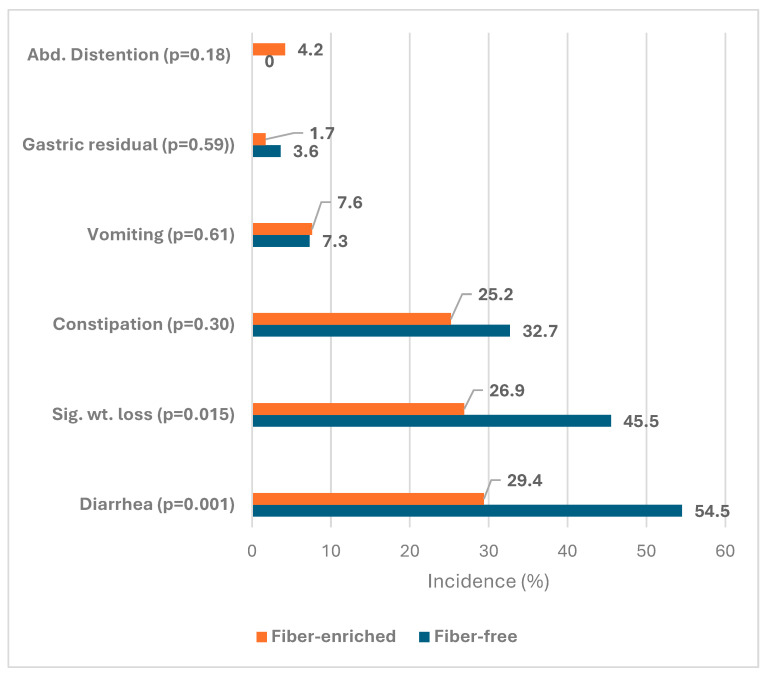
Incidence of bowel function complications in fiber-enriched and fiber-free groups of enteral feeding.

Table 2 shows the characteristics of patients in the FF and FE groups who received tube feeding and the incidence of complications. It shows that there was no significant difference between both groups in gender (*p* = 0.60), mean age (*p* = 0.059), mean weight (*p* = 0.051), mean BMI (*p* = 0.08), rate of underweight (*p* = 0.10), mean CCI score (*p* = 0.07), post ICU admission (*p* = 0.85), antibiotic medication (*p* = 0.17). or positive *C. difficilis* (*p* = 1.0). However, the FE group showed a significantly higher volume ratio (80.8% versus 73.1%, *p* = 0.009) and a significantly lower rate of laxative medication (77.3% versus 90.9%, *p* = 0.031) than the FF group. Regarding complications, the FE group showed a significantly lower incidence of diarrhea (29.4% versus 54.5%, *p* = 0.001) and significant weight loss (26.9% versus 45.5%, *p* = 0.015), Table 2 and Figure 1.

Table 3 shows the incidence of diarrhea for FF and Fe groups. The rate of diarrhea dropped from 54.5% for FF to 29% for FE groups, with an ARR of 25.1% (95% CI 24.6–25.6, *p* < 0.001) and an RRR of 64.1%. Relative risk was 0.54, reflecting a reduction in the rate of diarrhea by 46% after fiber enrichment. Concerning significant weight loss, the rate of significant weight loss dropped from 45.5% without enrichment to only 26.9% with enrichment, with an ARR of 18.6% (95% CI 18.0–19.2, *p* < 0.001) and an RRR of 40.9%. Relative risk was 0.59, reflecting a 41% reduction in significant weight loss after fiber enrichment.

Table 4 shows that, after adjusting for some potential confounders, fiber-free formula was a significant predictor of diarrhea (OR = 3.04, 95% CI 1.49–6.19, *p* = 0.002) and significant weight loss (OR = 2.37, 95% CI 1.16–4.84, *p* = 0.018) in tube feeding. Antibiotic intake was also a significant predictor of diarrhea (OR = 2.68, 95% CI 1.12–6.38, *p* = 0.026).

## 4. Discussion

In the present study, we relied upon more objective measures of the impact of fiber enrichment on the reduction of complications and the effect on bowel function. In this study, 46% and 41% reductions were observed in the incidence of diarrhea and significant weight loss. Even after adjusting for some potential confounders, the fiber-free formula was a significant predictor of diarrhea and significant weight loss in tube feeding. Patients on the fiber-free formula were three (and two) times more likely to contract diarrhea and significant weight loss than those on the fiber-enriched formula. Antibiotic intake was also a significant predictor of diarrhea, where patients who took antibiotics during tube feeding were two times more likely to experience diarrhea.

Diarrhea in enterally fed patients is a common problem, with a reported incidence ranging from 6% to 60% in one study [42] and 2% to 95% in others [14]. In our study, the incidence of diarrhea was 29.4% and 54.5% for the FE and FF groups, respectively. The wide-ranging incidence rate was attributed to the heterogeneity of the population and the lack of a standardized definition of diarrhea internationally. A recent and comprehensive meta-analysis has demonstrated a significant reduction in the percentage of patients with diarrhea when fed with a fiber-containing enteral feed (odds ratio of 0.68, with a 95% confidence interval of 0.46 to 0.95) [43]. In our study, the RRR of diarrhea was 46.1%, with a 46% reduction in the incidence of diarrhea. Patients on the fiber-free formula were three (OR = 3.04) more likely to contract diarrhea than those on the fiber-enriched formula. This may explain the favorable impact of fiber enrichment on patients’ quality of life while on tube feeding.

Several systematic reviews have demonstrated the positive effects of exclusive EN using FS feeds on the incidence of diarrhea and stool frequency in hospitalized, non-critically ill patients [32,43,44,45,46]. In our study, the FE group showed a significantly lower incidence of diarrhea (29.4% versus 54.5%, *p* = 0.001). Research evidence did not support direct links between enteral feeding and diarrhea and remained controversial [47]. Diarrhea can lead to feeding disruptions and complications such as electrolyte imbalance, dehydration, and increased vulnerability to wound infection, making it one of the most crucial issues to avert [48]. However, even after adjusting for confounders, the fiber-free formula significantly predicted diarrhea (OR = 3.04, *p* = 0.002) in tube feeding.

In clinical practice, diarrhea is attributed to infectious processes and antibiotic medication, protein–energy malnutrition, patient-related causes such as stress and surgical procedures, and bacterial gut contamination [49]. These conditions may compromise gut tolerance to enteral feeding, potentiating the risk of developing diarrhea. Diarrhea complicating enteral feeding is very common in all clinical settings. The major risk factor is using concomitant antibiotics. The most common association between diarrhea and enteral feeding remains the concomitant use of antibiotics. However, in our study, antibiotic medications and fiber-free enteral feeding were two independent risk factors for diarrhea. In our study, antibiotic intake was also a significant predictor of diarrhea (OR = 2.68, *p* = 0.026). There is no evidence that enteral feeding and antibiotics given concurrently act synergistically to increase the incidence of diarrhea, and it is probably the case that they are both risk factors acting independently of each other [42].

As reported in the literature, the weight loss mechanism in enteral feeding involves the rapid and direct delivery of nutrients to the jejunum [50,51]. This process stimulates the release of multiple appetite-suppressing gut hormones, changes nutrients and stretch sensing, activates the vagus nerve, and alters bile metabolism [52]. These physiological changes promote a fed state and reduce food intake, leading to weight loss [52]. In our study, the rate of significant weight loss dropped significantly from 45.5% without fiber enrichment to only 26.9% with enrichment, with an ARR of 18.6% and RRR of 40.9%. Relative risk was 0.59, reflecting a 41% reduction in significant weight loss after fiber enrichment. Patients on the fiber-free formula were two (OR = 2.37) times more likely to contract significant weight loss than those on the fiber-enriched formula.

## 5. Strengths and Limitations

The strength of this study is that it is the first prospective study in the region to tackle this issue of central feeding preference among non-critically ill patients. However, this study had some limitations. First, we could not guarantee the efficacy of the FE feeding due to the observational study design. Second, the heterogeneity of the patients, such as including patients with varying severity of illness, could confound the results. Third, diarrhea is attributed to other clinical practice confounders, including patient-related causes such as stress surgical procedures and bacterial gut contamination [49]. These conditions may compromise gut tolerance to enteral feeding, potentiating the risk of developing diarrhea. Fourth, our study did not investigate the physiological and clinical impacts of fiber-containing enteral formulas on diarrhea and bowel functions.

## 6. Conclusions

Overall, the study provides compelling evidence supporting fiber-enriched enteral feeding, though further discussion on potential confounders and clinical applications would enhance its impact. It demonstrated the efficacy of fiber supplementation in minimizing diarrhea and significant weight loss in non-critically ill patients receiving exclusive enteral tube feeding. However, fiber addition does not compromise nutritional efficiency, nor does it seem to offer a clinically evident metabolic advantage. Since controlling symptoms is significant in healthcare, it seems justifiable to recommend fiber-supplemented feedings as the standard tube-feeding formula. This advice should be based on the clinically perceptible positive effects of regulating colonic function rather than on the claimed nutritional and metabolic advantages, which remain to be proven.

Diarrhea management remains a combination of removing risk factors, such as concomitant antibiotics. Antibiotic usage must be scrutinized and stopped if possible. A well-designed RCT on non-critically ill patients is needed to prove the efficacy of fiber-enriched feeds used in enteral tube feeding, and its impact on bowel function, patient recovery, and hospital length of stay.

## Figures and Tables

**Table 1:** Contents of Ensure and Jevity formula per 1000 Calories.

Nutrition Information	Jevity	Ensure
Energy (Calories)	1060	1000
Protein (g)	44	40
Fat (g)	35	34
Carbohydrate (g)	155	137
Fiber (g)	14.4	0
Osmolality (mOsm/kg)	300	376

**Table 2:** Patient characteristics and incidence of complication in fiber-free and fiber-enriched groups of patients.

	Fiber-Free (FF) [*n* = 55]	Fiber-Enriched (FE) [*n* = 119]	Difference
*Patient characteristics*			
Age in years (mean ± SD)	50.0 ± 25.8	57.7 ± 21.9	t = 1.91, *p* = 0.059
Gender (m/f) (*n*)	36/19	73/46	X^2^ = 0.27, *p* = 0.60
Weight in Kg (mean ± SD)	59.2 ± 20.1	65.1 ± 14.5	t = 1.97, *p* = 0.051
BMI (mean ± SD)	22.9 ± 7.1	24.7 ± 5.7	t = 1.75, *p* = 0.08
Underweight (*n*, %)	21 (38.2)	31 (26.1)	X^2^ = 2.64, *p* = 0.10
CCI score (mean ± SD)	2.8 ± 2.5	3.6 ± 2.8	t = 1.83, *p* = 0.07
Post ICU admission (*n*, %)	8 (14.5)	16 (13.4)	X^2^ = 0.04, *p* = 0.85
Volume ratio (%)	73.1 ± 18.2	80.8 ± 17.6	t = 2.63, *p* = 0.009 *
Laxative medication (*n*, %)	50 (90.9)	92 (77.3)	X^2^ = 4.63, *p* = 0.031 *
Antibiotics administration (*n*, %)	6 (10.9)	23 (19.3)	X^2^ = 1.92, *p* = 0.17
*C. difficilis* + ve (*n*, %)	1 (1.8)	2 (1.7)	FET, *p* = 1.0
*Complications*			
Diarrhea (*n*, %)	30 (54.5)	35 (29.4)	X^2^ = 10.15, *p* = 0.001 *
Significant weight loss (*n*, %)	25 (45.5)	32 (26.9)	X^2^ = 5.89, *p* = 0.015 *
Constipation (*n*, %)	18 (32.7)	30 (25.2)	X^2^ = 1.06, *p* = 0.30
Vomiting (*n*, %)	4 (7.3)	9 (7.6)	FET, *p* = 0.61
Gastric residual (*n*, %)	2 (3.6)	2 (1.7)	FET, *p* = 0.59
Abdominal distention (*n*, %)	0 (0.0)	5 (4.2)	FET, *p* = 0.18
Regurgitation (*n*, %)	0 (0.0)	0 (0.0)	

FF—fiber-free group, FE—fiber-enriched group, CCI score—Charlton comorbidity score, X^2^—Pearson Chi-square test, t—student *t*-test, FET—Fisher-exact test, *—Significant difference.

**Table 3:** Risk reduction in diarrhea incidence and significant weight loss due to fiber enrichment in tube feeding.

**Diarrhea**	
Incidence of D+ in FF group (CER) %	54.5
Incidence of D+ in FE group (EER) %	29.4
ARR on D+ reduction % = (CER − EER) = 54.5 − 29.4	25.1 (95% CI 24.6–25.6)
RRR on D+ % = (CER − EER)/CER = (54.5 − 29.4)/54.5	46.1
Relative risk (RR) = EER/CER = 29.4/54.5	0.54
**Significant weight loss**	
Incidence of D+ in FF group (CER) %	45.5
Incidence of D+ in FE group (EER) %	26.9
ARR on D+ reduction % = (CER − EER) = 45.5 − 26.9	18.6 (95% CI 18.0–19.2)
RRR on D+ % = (CER − EER)/CER = 45.5 − 26.9)/45.5	40.9
Relative risk (RR) = EER/CER = 26.9/45.5	0.59

CER—control event rate, EER—experimental event rate, RR—relative risk, ARR—absolute risk reduction, RRR—relative risk reduction.

**Table 4:** Predictors of diarrhea and significant weight loss among patients on tube feeding.

	Diarrhea			Significant Weight Loss		
Predictors	B (SE)	*p*-Value	OR (95% CI)	B (SE)	*p*-Value	OR (95% CI)
Fiber free ^#^ versus fiber enrichment	1.11 (0.36)	0.002 *	3.04 (1.49–6.19) *	0.86 (0.36)	0.018 *	2.37 (1.16–4.84) *
Antibiotic medication (yes ^#^ versus no)	0.98 (0.44)	0.026 *	2.68 (1.12–6.38) *	−0.16 (0.48)	0.74	0.85 (0.33–2.19)
Laxative medication (yes ^#^ versus no)	0.20 (0.48)	0.68	1.22 (0.48–3.11)	−0.42 (0.47)	0.37	0.66 (0.26–1.64)
Volume ratio (%)	−0.01 (0.01)	0.50	0.99 (0.98–1.01)	−0.02 (0.01)	0.08	0.98 (0.96–1.00)
Constant	−0.69 (0.92)	0.45	0.50	0.64 (0.93)	0.49	1.89

# reference category, * statistically significant.

## Data Availability

Most of the data supporting our findings are contained within the manuscript, and all others, excluding identifying/confidential patient data, will be shared upon request.
